# Immunosuppressive mechanisms of oncofetal reprogramming in the tumor microenvironment: implications in immunotherapy response

**DOI:** 10.1042/BST20220157

**Published:** 2023-03-20

**Authors:** Jennifer Currenti, Archita Mishra, Michael Wallace, Jacob George, Ankur Sharma

**Affiliations:** 1 Harry Perkins Institute of Medical Research, QEII Medical Centre, 6 Verdun Street, Nedlands, WA 6009, Australia; 2 Curtin Medical School, Curtin University, 410 Koorliny Way, Bentley, WA 6102, Australia; 3 Neonatal Infection and Immunity, Telethon Kids Institute, 15 Hospital Ave, Nedlands, WA 6009, Australia; 4 Department of Hepatology and Western Australian Liver Transplant Service, Sir Charles Gairdner Hospital, Hospital Ave, Nedlands, WA 6009, Australia; 5 Medical School, University of Western Australia, 35 Stirling Hwy, Nedlands, WA 6009, Australia; 6 Storr Liver Centre, Westmead Institute for Medical Research, Westmead Hospital and University of Sydney, 176 Hawkesbury Rd, Westmead, NSW 2145, Australia; 7 Institute of Molecular and Cellular Biology (IMCB), Agency for Science, Technology and Research (A*STAR), Singapore; 8 KK Research Centre, KK Women's and Children's Hospital, 100 Bukit Timah Rd, 229899 Singapore

**Keywords:** cancer, immunosuppression, immunotherapy, oncofetal, tumor microenvironment

## Abstract

Both fetal and tumor tissue microenvironments display immunosuppressive features characterized by the presence of specific immunomodulatory stromal and immune cell populations. Recently, we discovered shared microenvironments between hepatocellular carcinoma (HCC) and fetal tissues and described this phenomenon as an oncofetal ecosystem. This ecosystem includes fetal-like immune (macrophage) and stromal (endothelial) cells within the tumor microenvironment (TME). This discovery highlights reciprocal interactions between fetal-like macrophages and T cells which result in the orchestration of an immunosuppressive TME. Importantly, VEGF-A protein expression by tumor cells and fetal-like macrophages plays an important role in oncofetal reprogramming of the TME in HCCs. Interestingly, recent clinical data indicate that blocking VEGF-A or CTLA4 alongside PD-L1 is effective in treating advanced HCC. Consequently, some immunotherapies may target and rely on oncofetal cells for clinical responsiveness. This understanding provides exciting opportunities to utilize oncofetal niche characteristics as biomarkers of immunotherapy response in HCC and might also have validity for predicting responses to immunotherapy in other cancers. In this review, we explore the immunosuppressive mechanisms and interactions of oncofetal cells in the TME of HCC and their potential implications for immunotherapy response.

## Introduction

Immunosuppression is characterized by a reduction in the efficacy and activation of immune responses [1]. Two illustrations of immunosuppression are during pregnancy/fetal development and within the tumor microenvironment (TME) [2–7]. Many elements of the immune system are immunosuppressive by trait and can exert immunosuppressive effects on other components of the immune system [8]. These include regulatory T cells (Tregs) [9], mature DCs enriched in immunoregulatory molecules (mregDCs) [3,7], regulatory B cells (Breg) [10], and immunosuppressive macrophages [11] to name a few. These regulatory cells may be involved in both tumor progression [2,3] and fetal development [4–7]. Fetal development requires immunosuppression within both the mother and the fetus for healthy fetal development [12–15], whereas immunosuppression within the TME is a determinant for the survival of tumor cells [16].

Oncofetal reprogramming describes the appearance of characteristics reminiscent of fetal development in tumor cells and more recently, the TME [17]. Tumor development was linked to embryonic development in the early 1900s [18,19] where it was hypothesized that tumors were an abnormal extension of embryonic cells [18]. This theory underwent several permutations over the decades [18,20–24] and recently, oncofetal reprogramming has been extended beyond tumor cells to encompass cells of the TME in hepatocellular carcinoma (HCC), termed the oncofetal ecosystem [17]. The oncofetal ecosystem in HCC is characterized by a VEGF-A and PD-L1 mediated immunosuppressive microenvironment that exists in tumor and fetal tissues but is absent from normal adult liver tissue [17,25,26]. Consequently, oncofetal cells within the TME can impact immunosuppressive features and are likely targeted by immunotherapies due to overlapping targets.

The use of immune checkpoint inhibitors (ICIs) in cancer has been transformative in the treatment of several malignancies, including melanoma [27,28], renal-cell carcinoma [29], bladder cancer [30], non-small lung cell cancer [31], and others [32–34]. Furthermore, ICIs target immunosuppressive receptors and ligands (primarily PD-1, PD-L1, CTLA4, and LAG-3) with the aim to reduce immunosuppression within the TME [32,35–39]. While ICIs have been revelatory in the treatment of several malignancies, there are cancers where ICIs have had limited success, for example, HCC [40]. In this setting of HCC, the recently discovered role of VEGF-A and PD-L1 mediated immunosuppression by oncofetal cells within the TME [17] suggests that these cells may be implicated in ICI response [41]. Consequently, this review aims to delve into the mechanisms of immunosuppression in fetal development and tumorigenesis and explore the potential implications of such mechanisms in cancer therapy with a focus on HCC.

## Immunosuppression in early development and cancer

### Immunosuppression during fetal development

Fetal growth and development represent the best and most successful examples of graft-tolerance in the body with any disruption in this tolerance leading to pre-term birth or altered fetal growth [42]. The fetus shares only half of its genes with the mother and an array of non-inherited maternal antigens and several rapidly evolving self-antigens always pose a risk for immune intolerance [14]. Yet although most of the immune cells and both innate and adaptive components of immunity are present in the fetus, maternal–fetal exchanges do not mount an immune response [7]. One reason could be the selective barrier between the two; however, this does not protect the fetal environment from antigenic and/or metabolic exposures [43]. Research has shown that the fetal environment is protected by regulatory immune cells that calibrate and balance the immune responses generated by the fetus [7,44,45]. This is a delicate balancing act where the immune system is not only protecting the fetus by virtue of tolerance, but also constantly learning and evolving to be primed for exposures after birth. For optimal development, this requires continuous communication between immune cells and the surrounding environment.

An immunosuppressive environment during fetal development is critical for maternal–fetal homeostasis [13]. Maternal–fetal tolerance during pregnancy is essential and is characterized in the mother by a shift in immune cell populations to mediate immune-endocrine interactions [46]: an increase in Tregs and a shift from Th1 to Th2 responses [13,47] or more simply, a pro-inflammatory to an anti-inflammatory phenotype. While this occurs in the mother to prevent maternal T cells attacking the fetus [48], the fetal adaptive immune system also generates Tregs to suppress the function of fetal T cells specific for maternal alloantigens [4]. Maternal cells also cross the placenta to engraft *in utero* into fetal tissues, a phenomenon known as maternal microchimerism [49]. Because of this, fetuses display elevated proportions of Tregs in lymphoid tissues compared with adults [50]. The crucial role of fetal Tregs in maintaining maternal–fetal tolerance is further highlighted by their decline at birth [51]. This is likely due to differing cytokine environments; increased expression of transforming growth factor (TGF) β family members has been observed in fetal lymph nodes compared with adults, with TGFβ signaling critical for Treg differentiation during T cell activation [4]. While the expression of TGFβ family members plays a large role in fetal development [52], it also promotes a favourable microenvironment for tumor growth [53].

Fetal dendritic cells (DCs) have been shown to migrate to lymph nodes to promote tolerogenic immunity through Arginase-2-induced Treg maintenance [7]. Similarly, CD71^+^ erythroid suppressor cells are present in the fetus and enhance regulatory T cell-mediated immunosuppression through Arginase-2 activity [54,55]. Conversely, dysfunctional Treg cells have been implicated in pre-term birth, miscarriages, pre-eclampsia, and inability for embryo-implantation [56–58]. Interestingly, the adoptive transfer of Tregs in mice with recurrent miscarriages helps establish successful pregnancy and fetal development [59]. These observations strongly indicate the importance of maintaining an immunosuppressive environment during development and illustrates the complicity of common signaling and metabolic pathways to achieve this goal. Just as the fetal environment constantly evolves, so has knowledge of early life development and immune-tolerance mechanisms that work toward a successful maternal–fetal association.

### Immunosuppression in tumors

An immunosuppressive microenvironment is critical for tumor development, but tumor cells also drive the generation, expansion, and recruitment of immunosuppressive cell types in the TME [60,61]. In HCC, primary tumors commonly reside in inflamed liver tissue [62], with macrophages playing a role in the initiation of HCC in inflamed livers [63]. Tumor-associated macrophages (TAMs) compose a significant proportion of the HCC TME [64] and secrete chemokines which attract Tregs to the TME, thereby enhancing immunosuppression [65]. Furthermore, TAMs and FoxP3^+^ Tregs were found to co-localize and promote HCC progression [66]. This recruitment of immunosuppressive cells acts in part to establish an immunosuppressive TME while the sequestration of cells results from the expression of immunosuppressive cytokines such as vascular endothelial growth factor (VEGF) and TGF by tumor cells in several cancers [67], including HCC [17,68]. Consequently, immunosuppressive cells play a significant role in the development of primary HCC [62]. This has been similarly reported in colorectal cancer where modeling predicted that the recruitment of immunosuppressive cells was the most common driver of benign to malignant transformation [69].

The incidence of cancer increases with advancing age [70], as does the deterioration of the immune system (termed immunosenescence) [71], suggesting a potential association of the two. Immunosenescence is primarily described with respect to T cells in elderly individuals, largely due to the maintenance of antigen presentation by DCs (one of the major antigen-presenting cells) [72]. However, alongside immunosenescence is an increase in inflammation, termed inflammaging [73]. In some cases, immunosuppressive cells are induced by inflammatory mediators [74,75] including NFκB, the STAT family transcription factors [76,77], chemokines, and colony-stimulating factors [75] (such as inflammaging in the elderly and chronic inflammation in HCC). Therefore, the immunosuppressive TME orchestrated by cells in the TME of HCC is likely related to inflammation. Furthermore, the immune system of the aged somewhat resembles that of a newborn: compromised lymphocyte responses, reduced activity of macrophages and neutrophils, decreased natural killer (NK) cell killing, and reduced antigen presentation by DCs [78]. Consequently, immunosuppression is characteristic of both fetal development and tumorigenesis with overlapping immunosuppressive cells driving these microenvironments.

## Immunosuppressive nature of oncofetal cells in the TME

An immunosuppressive microenvironment is characteristic of fetal development and tumorigenesis and is an important component of tumor progression and metastasis [17,67,68]. Several cells within the TME have been shown to display oncofetal reprogramming, the most well-described being macrophages [11,17] and endothelial cells [17,79]. Importantly, oncofetal reprogramming has been discovered in a subset of the TME population in HCC and further research in this direction will shed light on new oncofetal cell types in the tumor ecosystem. Therefore, in this review, we will discuss the potential role of an oncofetal ecosystem in immunotherapy response.

### FOLR2^+^ oncofetal macrophages

Recently, we discovered the re-emergence of fetal-like macrophages in the microenvironment of HCC and highlighted their implication in the immunosuppressive microenvironment [17]. Folate receptor beta (FOLR2)-positive macrophages first emerge in the yolk sac and early fetal organs, with fate-mapping of adult samples indicating emergence from both yolk sac and fetal monocyte precursors, reflective of their embryonic origins [80]. During fetal development, fetal and maternal-derived macrophages are observed in the placenta, playing niche-specific roles vital for normal placental development and function [5]. Fetal-derived macrophages (Hofbauer cells; HBC) in the first-trimester express (and are defined by) the *FOLR2* gene [45] and interestingly, are the only immune cells found within the stromal core at this point [5]. In contrast, placenta-associated maternal monocytes/macrophages do not express FOLR2 [5], indicating that FOLR2 expression is specific to fetal development in this context. Importantly, FOLR2^+^ HBCs/macrophages [45,81], as well as some tumor cells [17,82], are the main source of VEGF in tissue ecosystems, thus contributing toward angiogenesis in fetal and tumor development, such as in HCC [17]. VEGF secreted by both HBCs in the placenta and tumor cells target endothelial cells (ECs) [5]. Consequently, VEGF signaling plays a role in angiogenesis in both fetal [5] and tumor [83] development, and in HCC VEGF signaling results in the emergence of oncofetal ECs [17].

TAMs have recently been shown to express FOLR2 in HCC and breast cancer [11,17,84,85]. The TME contains ontogenically distinct TAM populations; one population arises from embryonic resident tissue macrophages pre-existing in the tissue prior to tumor development, and the second population arises from inflammatory monocytes recruited from the circulation [86,87]. Interestingly, in tumor tissues, FOLR2^+^ TAMs are derived from both embryonic and adult bone marrow precursors, demonstrating the ability for adult macrophages to be reprogrammed into a fetal-like state, regardless of ontogeny [11,17].

**Figure 1.**
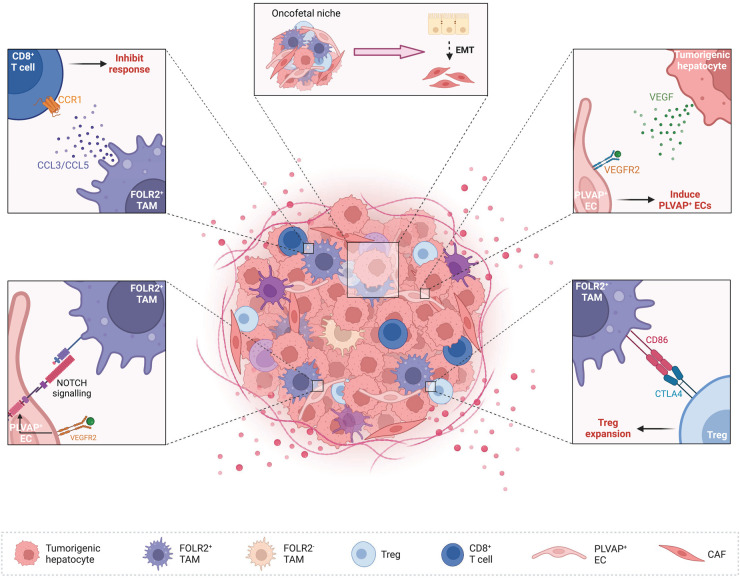
Oncofetal cells within the TME promote immunosuppression. Tumorigenic hepatocytes and FOLR2^+^ TAMs secrete VEGF within the TME of HCC, inducing the oncofetal reprogramming and expansion of PLVAP^+^ ECs, with NOTCH signalling maintaining the oncofetal ecosystem. Furthermore, high expression levels of CD86 by FOLR2^+^ TAMs induces Treg expansion. To further enhance the immunosuppressive environment, cytokines and chemokines secreted by FOLR2^+^ TAMs inhibit the cytotoxic response of T cells. An immunosuppressive TME orchestrated by oncofetal cells provides the basis for EMT and metastasis. Created with BioRender.com.

FOLR2^+^ TAMs play a role in creating an immunosuppressive TME. For example, FOLR2 is preferentially expressed by macrophages with anti-inflammatory properties [88] and FOLR2^+^ TAMs inhibit both cytokine secretion and the proliferation of tumor-specific T cells [89]. Furthermore, high expression levels of immunomodulatory chemokines and CD86 by FOLR2^+^ TAMs further support their likely role in facilitating an immunosuppressive TME [17]. In the case of HCC, cell–cell interaction analyses identified FOLR2^+^ TAMs as having more immunosuppressive interactions with Tregs in comparison with other TAM populations within the TME [17]. Similarly, NOTCH signaling between FOLR2^+^ TAMs and ECs worked in synergy with VEGF signaling in the TME of HCC, promoting the maintenance of an oncofetal ecosystem [17]. However, the co-localization of fetal-like FOLR2^+^ TAMs with Tregs and fetal-like ECs adjoining ALB^+^ tumor epithelial cells in HCC suggests that they do not orchestrate an immunosuppressive TME alone [17] (Figure 1).

### PLVAP^+^ oncofetal endothelial cells

Alongside FOLR2^+^ TAMs, we identified fetal-like ECs and their implications for maintaining the oncofetal ecosystem in the TME of HCC [17]. Plasmalemma vesicle-associated protein (PLVAP)-positive endothelial cells have been identified in fetal [90,91], cirrhotic liver [92], and HCC [17,93], and are enriched in the tumor periphery [17]. PLVAP is an EC-specific gene involved in angiogenesis, leukocyte migration, and basal permeability [90,91]. Furthermore, PLVAP^+^ cells control the accumulation of tissue-resident fetal liver macrophages in a selective manner but do not impact yolk sack- or bone marrow-derived macrophages or other leukocytes [91]. In a murine model, adult *Plvap^−/−^* mice displayed significantly decreased levels of tissue-resident embryonic liver-derived macrophages in the spleen, peritoneal cavity, and lungs [91]. Consequently, it is thought that PLVAP^+^ ECs in the TME may facilitate reprogramming to fetal-like macrophages [17], subsequently, promoting an immunosuppressive microenvironment. Alongside FOLR2^+^ macrophages, PLVAP^+^ ECs have been identified in a gastric TME [94].

In a murine model of HCC, VEGF expression from malignant hepatocytes induced PLVAP expression in liver sinusoidal ECs (LSECs), in turn promoting FOLR2^+^ TAMs and interactions with Tregs [17]. In this murine model, PLVAP^+^ LSECs regulated the entry of lymphocytes and antigens into lymph nodes [90]. Furthermore, LSECs can impact liver disease outcomes such as portal hypertension, fibrosis and autoimmune hepatitis, and may induce immune tolerance in the liver [95]. Combined, PLVAP^+^ LSECs have the capacity to impact immunosuppression in fetal and tumor tissue. VEGF/NOTCH signaling is also an important aspect of oncofetal reprogramming [17], with VEGF expression shown to induce PLVAP^+^ ECs [17] and in turn, FOLR2^+^ TAMs (Figure 1). These interactions are suggestive of an immunosuppressive communication hub inclusive of LSECs in the liver TME that can facilitate tumor growth [95].

## Potential implication of FOLR2^+^ oncofetal macrophages in immunotherapy response

Several immunotherapies for the treatment of cancer are aimed at reversing and/or combatting immunosuppression in the TME. Tumor-infiltrating immunosuppressive cells exploit inhibitory molecules to impair T cell-mediated responses [35]. Consequently, immune checkpoint inhibitors (ICIs) target these checkpoints (such as PD-1, PD-L1, CTLA4, and LAG-3) to block checkpoint receptor–ligand interactions, permitting a robust cytotoxic lymphocyte response [96]. The context of immune cells in the tumor ecosystem impacts the efficacy of ICIs and as immunotherapies only work in a fraction of cancer patients, there is a critical need to identify biomarkers and new targets/combinations to treat non-responding tumors.

Therapies combining an anti-angiogenic and an ICI have resulted in breakthroughs for the treatment of unresectable HCC [97,98], advanced renal-cell carcinoma [99,100], metastatic non-small-cell lung cancer [101], and endometrial carcinoma [102]. In 2020, a new combination of an anti-VEGF-A inhibitor (bevacizumab) and an anti-PD-L1 (atezolizumab) demonstrated significant efficacy and became the standard-of-care for HCC based on the results of IMbrave150 [97,103]. This combinatorial therapy of an ICI with an anti-angiogenic antibody drives the infiltration of immune cells into ‘cold’ tumors, thus converting them to ‘hot’ tumors with the goal of increasing therapy response [104]. Simply, the synergy of the combinatorial treatment modulates blood supply to the tumor and enhances the anti-tumor immune response [97], reducing immunosuppression and harnessing the TME of HCC to eliminate tumor cells. More recently, the combination of anti-PD-L1 (durvalumab) and anti-CTLA4 (tremelimumab) has been approved by the US FDA for use in unresectable HCC based on the results of HIMALAYA [105]. However, these combinatorial therapies only have an objective response rate of 30% [40] and 20.1% [105] for IMbrave150 and HIMALAYA, respectively. Consequently, there is still a need to identify therapies for the remaining HCC patients who do not respond or who become resistant to therapy. As such, there are several ongoing and upcoming clinical trials for immunotherapies in HCC (reviewed in [106]), many of which have the potential to target oncofetal cells in the TME (Table 1).

**Table 1.** **Approved, current, and upcoming ICI therapies for HCC that may target oncofetal** **cells in the TME**

Trial name	Identifier	Phase	BCLC stage*	Treatment arms^†^	Setting
CheckMate 040	NCT01658878	Approved		Nivolumab (**anti-PD-1**) single arm	Second
CheckMate 040	NCT01658878	Approved		Nivolumab (**anti-PD-1**) + ipilimumab (**anti-CTLA4**) single arm	Second
IMbrave150	NCT03434379	Approved		Atezolizumab (**anti-PD-L1**) + bevacizumab (**anti-VEGF**) vs. sorafenib	First
CheckMate 9DW	NCT04039607	Phase 3	C	Nivolumab (**anti-PD-1**) + ipilimumab (**anti-CTLA4**)	First
				Sorafenib or lenvatinib	
HIMALAYA	NCT03298451	Phase 3	B or C	Durvalumab (**anti-PD-L1**)	First
				Durvalumab (**anti-PD-L1**) + tremelimumab (**anti-CTLA4**) 2 regimens	
				Sorafenib	
N/A	NCT03764293	Phase 3	B or C	Camrelizumab (SHR-1210; **anti-PD-1**) + apatinib (**anti-VEGFR2**)	First
				Sorafenib	
Bayer 19497	NCT03347292	Phase 1b/2	B or C	Regorafenib (**anti-VEGFR**) + pembrolizumab (**anti-PD-1**)	First
GOING	NCT04170556	Phase 1/2	BCLC C	Regorafenib (monotherapy for the first 8 weeks; **anti-VEGFR**) + nivolumab (**anti-PD-1**)	Second
ORIENT-32	NCT03794440	Phase 2/3	B or C	Sintilimab (**anti-PD-1**) + IBI305 (**anti-VEGF**)	First
				Sorafenib	
RENOBATE	NCT04310709	Phase 2	B or C	Regorafenib (**anti-VEGFR**) + nivolumab (**anti-PD-1**)	First
N/A	NCT04183088	Phase 2	B or C	Part 1:	First
				Regorafenib (**anti-VEGFR**) + tislelizumab (**anti-PD-1**)	
				Part 2:	
				Regorafenib (**anti-VEGFR**) + tislelizumab (**anti-PD-1**)	
				Regorafenib (**anti-VEGFR**)	
N/A	NCT04442581	Phase 2	B or C	Cabozantinib (**anti-VEGFR**) + pembrolizumab (**anti-PD-1**)	First

*BCLC (Barcelona Clinic Liver Cancer) staging system is used to determine treatment [131]. It considers the number and size of liver tumors, liver function, and general health and fitness. Stage B is intermediate with several tumors in the liver, but liver function remains. Stage C is advanced where the metastases are present, but the liver is still functioning. ^†^ICI targets have been indicated in brackets with those implicated in the oncofetal ecosystem in bold.

**Figure 2.**
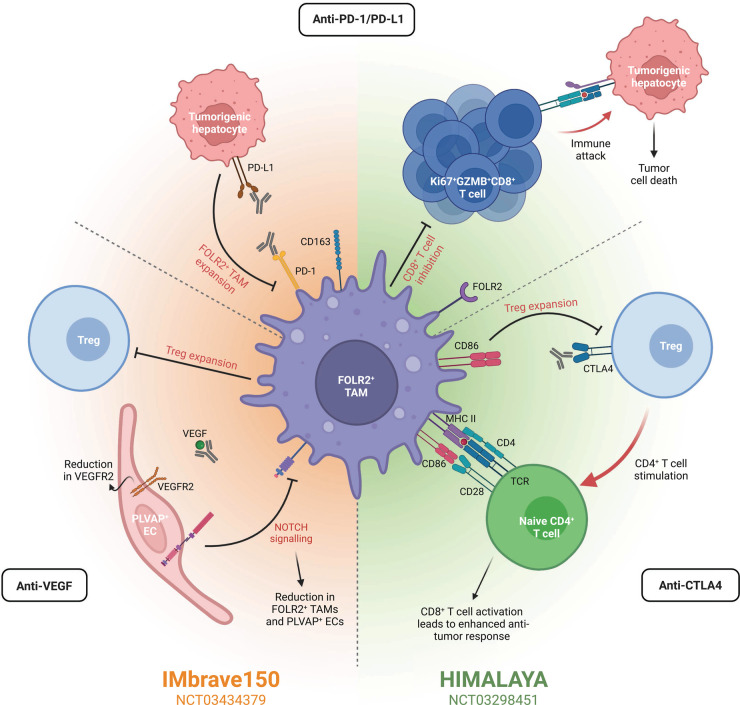
Oncofetal cells are implicated in immunotherapy response. Immunotherapies for HCC are often combinatorial, including the three main targets anti-PD-1/PD-L1, anti-VEGF, and/or anti-CTLA4. Two prominent clinical trials for HCC, IMbrave150 and HIMALAYA, assess the combination of these targets. The results of IMbrabe150 have led to the combination of anti-PD-L1 and anti-VEGF as the standard-of-care for advanced-stage HCC. Similarly, HIMALAYA is assessing the efficacy and safety of an anti-PD-L1 and anti-CTLA4. Pathways targeted by these therapies often include those implicated in oncofetal reprogramming or oncofetal cells directly. In the case of anti-PD-1/PD-L1 therapies, these can hinder the development of new immunosuppressive FOLR2^+^ TAMs by inhibiting the PD-1/PD-L1 axis. In turn, the inhibition of CD8^+^ T cells by FOLR2^+^ TAMs is reduced, permitting the proliferation of Ki67^+^GZMB^+^CD8^+^ T cells and resultant tumor cell death. Combination of an ICI with anti-VEGF, as in the IMbrave150 trial, further reduces the presence of FOLR2^+^ TAMs and PLVAP^+^ ECs in the TME. Anti-VEGF inhibits NOTCH signaling, reducing the presence of both FOLR2^+^ TAMs and PLVAP^+^ ECs. Furthermore, Treg expansion is also inhibited, diminishing the immunosuppressive microenvironment. Combining anti-PD-L1 instead with anti-CTLA4 therapies, as in the HIMALAYA trial, limits Treg expansion by eliminating the CTLA4-CD86 interaction. As such, CD86 is now able to provide the necessary co-stimulation to activate naïve CD4^+^ T cells and in turn, CD8^+^ T cell activation leads to an enhanced anti-tumor response. Consequently, the presence and targeting of oncofetal cells/reprogramming by immunotherapy within the TME may impact treatment response. Created with BioRender.com.

Macrophages are abundant in the TME of HCC and exhibit a heterogeneous nature [107], with FOLR2^+^ TAMs playing an important role in orchestrating an immunosuppressive TME [17]. FOLR2^+^ TAMs are ontogenically either embryonic macrophages or could be reprogramed monocyte-derived macrophages [17]. Cell–cell interaction analyses have suggested that FOLR2^+^ TAMs may be drivers of immunosuppression in HCC due to their increased expression of immunomodulatory chemokines alongside a greater number of immunosuppressive interactions with Tregs in comparison with other TAM populations [17]. Furthermore, an enrichment of FOLR2^+^ TAMs co-localized with Tregs and PLVAP^+^ ECs was observed compared with other TAM populations [17]. For these reasons, we postulate that interactions between FOLR2^+^ TAMs co-localized with Tregs, fetal-like ECs, and ALB^+^ tumor epithelial cells could be impacted by anti-angiogenic therapies (Figure 2), thereby, potentially impacting immunotherapy response. However, it is important to note that tumors display high levels of heterogeneity both within and between patients [93] and as such, the mechanisms speculated herein likely display spatial localizations and/or vary between tumors.

### Potential implications of anti-VEGF therapy on macrophage reprogramming

VEGF-mediated immunosuppression, driven by the overexpression of VEGF largely by malignant hepatocytes, has been implicated in HCC development and progression [108,109]. A recent study assessing the molecular mechanisms behind the combinatorial therapy of atezolizumab (anti-PD-L1) plus bevacizumab (anti-VEGF-A; IMbrave150) identified a reduction in VEGFR2 following treatment with atezolizumab and bevacizumab in comparison with atezolizumab alone, regardless of treatment response [41]. Furthermore, this study further identified a reduction in Treg signatures in responders following this combinatorial therapy; a mouse model suggests that this is likely due to a reduction in proliferating Tregs following atezolizumab plus bevacizumab or anti-VEGF alone (Figure 2) [41]. Similarly, the same mouse model showed a reduction in monocyte-derived macrophages and an increase in the number of CD8^+^ T cells following combinatorial therapy [41]. Combined, these observations highlight the potential for anti-VEGF to augment anti-tumor immunity in HCC [41], likely in part by impacting the oncofetal reprogramming of TAMs.

We speculate that a reduction in oncofetal reprogramming, and subsequently immunosuppression, within the TME may lead to an increased likelihood of therapy response. In support of this theory, VEGF is a well-described immunosuppressive cytokine [67] and serves to induce the oncofetal reprogramming of ECs and in turn TAMs in HCC [17]. As FOLR2^+^ TAMs have been shown to be modulators of immunosuppression in HCC [17], it is plausible that impacting their oncofetal reprogramming may lead to reduced immunosuppression. While oncofetal reprogramming has been demonstrated, it is likely to occur in a subpopulation of cells and may be subject to spatial and patient heterogeneity. As such, treatment with bevacizumab likely impacts the oncofetal reprogramming of PLVAP^+^ ECs and FOLR2^+^ TAMs due to the reduction in VEGF in patients whose tumors express high levels of VEGF. Moreover, subsequent downstream abrogation of NOTCH signaling could abrogate oncofetal reprogramming in HCC (Figure 2). Consequently, for tumors (HCC) with high levels of oncofetal cells and VEGF expression, the impact of anti-VEGF-A is likely to be more apparent due to their role in oncofetal reprogramming and orchestrating an immunosuppressive TME.

Aside from the implications of anti-VEGF therapy on macrophage reprogramming, VEGF plays a suppressive role in T cells [110] with VEGFR2 expressed by FOXP3 high Tregs [111]. VEGF has been shown to promote the proliferation of Tregs with VEGF/VEGFR2 blockade inhibiting such proliferation in colorectal cancer [112] (Figure 2). Therefore, anti-VEGF therapy may indirectly block the oncofetal reprogramming of FOLR2^+^ oncofetal TAMs alongside PLVAP^+^ ECs and reduce Treg expansion.

### Anti-CTLA4 therapy may impact Treg expansion by influencing FOLR2^+^ TAM-Treg interactions

CTLA4 (cytotoxic T-lymphocyte associated protein 4) is a receptor present on T cells and a critical inhibitor of T cell expansion [113,114], activation, and proliferation [115]. Anti-CTLA4 therapy can be used in place of, or following, anti-angiogenics (such as anti-VEGF) and critically, where anti-angiogenic therapy has led to resistance via the up-regulation of other pro-angiogenic factors or angiogenic signaling pathways [116]. Expression of CTLA4 is primarily by activated and regulatory T cells [117] and is critical for the direct and indirect immunosuppressive properties of Tregs [118,119]. FOLR2^+^ oncofetal TAMs are known to interact with Tregs through the CD86-CTLA4 axis in HCC (Figure 2) [17]. Stimulation of Tregs by these fetal-like FOLR2^+^ TAMs is anticipated to be abrogated by anti-CTLA4 therapy, with CD86 instead able act as co-stimulation for the activation of naïve T cells and a subsequently enhanced anti-tumor response (Figure 2). This is supported by an increase in the abundance of CD4^+^ and CD8^+^ T cells, alongside increased T cell receptor diversity, following anti-CTLA4 therapy in HCC [120]; increased abundance of T cells occurs from restoring the balance between regulatory and effector compartments within the TME [113,121]. As such, we hypothesize that anti-CTLA4 agents will negatively impact the ability for fetal-like FOLR2^+^ TAMs to induce the expansion of Tregs in HCC, subsequently reducing the immunosuppressive TME and increasing the likelihood of therapy response.

### PD-1/PD-L1 expression in fetal-like FOLR2^+^ TAMs

Several cells in the TME are negatively regulated by PD-1 expression, including B cells, T cells [122], NK cells [123], DCs [124], and macrophages [125]. Overexpression of the corresponding ligand, PD-L1, frequently occurs in tumor cells and macrophages to facilitate escape from the immune response [126,127]. Due to this expression of PD-L1 on tumorigenic hepatocytes and the subsequent induction of fetal-like FOLR2^+^ TAMs [17], anti-PD-1/PD-L1 therapies may also impact FOLR2^+^ oncofetal TAMs (Figure 2); TAMs also express PD-L1 and resultantly, the use of anti-PD-L1 will blunt the functional impact of FOLR2^+^ TAMs. As such, patients with a high proportion of fetal-like TAMs expressing PD-L1 may benefit from treatment with an anti-PD-1/PD-L1. Such treatment may increase the likelihood of immunotherapy success in these patients by reducing immunosuppression within the TME. In support of anti-PD-1 therapy for HCC patients with oncofetal FOLR2^+^ TAMs expressing PD-L1, a murine model of PD-L1 knockout tumors indicated that PD-1–PD-L1 antagonism enhances the anti-tumor efficacy of myeloid cells, with this pathway inhibiting TAM function [125]. Furthermore, PD-1/PD-L1 blockade reduces tumor size with a direct influence on TAMs [125]. In a mouse model of atezolizumab plus bevacizumab, proliferating (Ki67^+^) antigen-specific CD8^+^ T cells were observed following combinatorial therapy as compared to bevacizumab or sorafenib alone (Figure 2) [41]. Furthermore, these CD8^+^ T cells expressed GZMB [41], indicating cytotoxic potential. As FOLR2^+^ oncofetal TAMs inhibit the response of CD8^+^ T cells in HCC [17], this observation of atezolizumab suggests it likely acts to reverse inhibition by TAMs and may be reflected following durvalumab, an anti-PD-L1. Hence, we speculate that FOLR2^+^ oncofetal TAMs may influence immunotherapy outcome.

## Precision medicine: determining the right combinatorial immunotherapy

**Figure 3.**
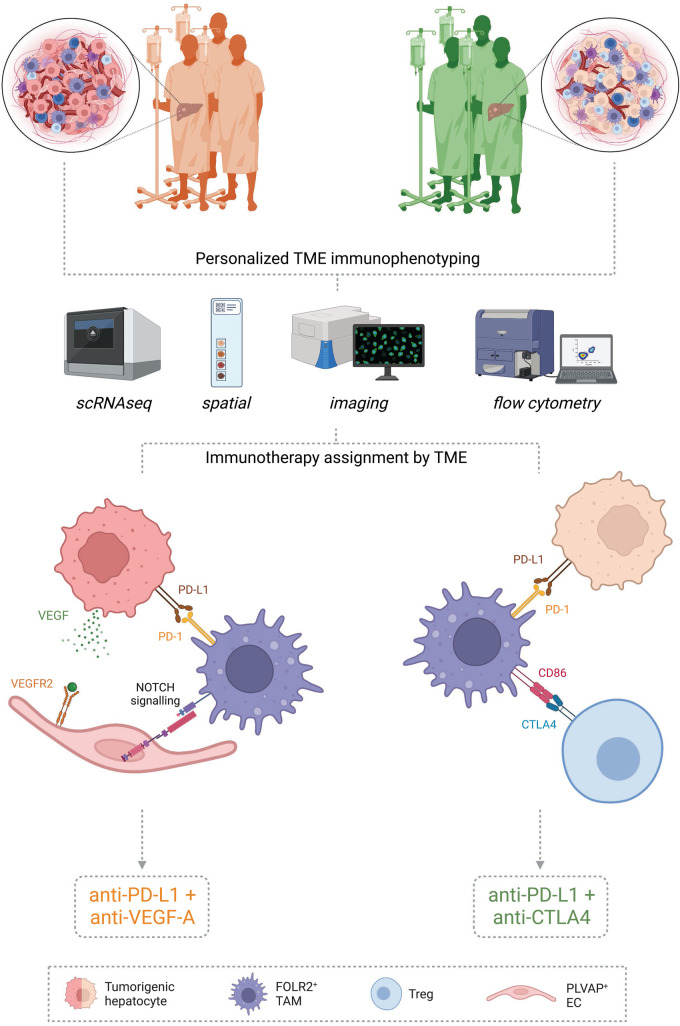
Tumor microenvironment characterization may guide personalized immunotherapy selection. Tumor tissue from patients to receive immunotherapy can be characterized by several means such as single-cell RNA sequencing (scRNAseq), spatial technologies, imaging, and/or flow cytometry to name a few. These technologies permit the determination of cell presence/quantification and cell–cell interactions that may be harnessed to determine which immunotherapy is likely to have the highest success rate for an individual tumor. Tumors with high PD-1/PD-L1 expression likely benefit from an anti-PD-1/PD-L1. Those with abnormal vasculature and high VEGF expression likely display oncofetal reprogramming for ECs and TAMs and will likely respond to an anti-VEGF. Conversely, an anti-CTLA4 is likely beneficial in tumors with an abundance of Tregs displaying CTLA4 expression. Consequently, immunophenotyping individual tumors may guide clinicians in the assignment of immunotherapies believed to be most likely to succeed. Created with BioRender.com.

We speculate that due to the importance of PD-1/PD-L1, CTLA4, and VEGF in both the oncofetal ecosystem and immunosuppression, their presence may be indicative of immunotherapy response and guide clinical decision making (Figure 3). This is exemplified by the co-localization of the oncofetal ecosystem and associated signaling pathways (VEGF-A and PD-L1) in the early HCC microenvironment [17]. Yet while IMbrave150 and HIMALAYA improve the median survival of patients with advanced HCC compared with sorafenib alone [97,128], their respective objective response rates of 30% [40] and 20.1% [105] exemplify the need to predict the outcome for a range of immunotherapies, enabling clinicians to customize immunotherapy decision making to a patients individual tumor(s).

Assignment of therapy may occur based on the expression of several markers and/or predominant cell–cell interactions as determined by single-cell RNA sequencing, spatial technologies, or other immunophenotyping assays performed on tumor samples (biopsy/resection; Figure 3). Tumors with a large proportion of fetal-like FOLR2^+^ TAMs and PD-1/PD-L1 expression may benefit from anti-PD-1/PD-L1 therapy (IMbrave150 or HIMALAYA). Tumors with high VEGF expression, or ‘cold’ tumors displaying abnormal vasculature, will likely benefit from an anti-angiogenic such as anti-VEGF, potentially in combination with an anti-PD-L1 as in IMbrave150. We speculate that the combination of an anti-VEGF and anti-PD-L1 will likely be beneficial in this context due to the implications on macrophage reprogramming and immunosuppression, as detailed above. Conversely, ‘hot’ tumors and/or those with VEGF resistance may see benefit from an anti-CTLA4 as in HIMALAYA, namely where an increased proportion of Tregs displaying CTLA4 is present. In summary, if the presence and extent of an oncofetal ecosystem provide the ability to predict immunotherapy usage and response, it will pave the way for precision medicine and may reduce the incidence of recurrence if used in an adjuvant setting for early stage HCC. This is illustrated by the promising results of IMbrave050, a Phase III global, multicenter, open-label, randomized study assessing the impact of anti-PD-L1 and anti-VEGF on recurrence when given as an adjuvant for early-stage HCC [129]; interim results showed a significant improvement in recurrence-free survival using this combinatorial immunotherapy post resection or ablation [130].

## Outlook: open questions surrounding oncofetal reprogramming

The TME is highly dynamic and heterogeneous, playing varied roles and having distinct characteristics as tumors progress. Interactions between cells of the TME provide the necessary microenvironment for tumor growth, with one important feature being immunosuppression [17,67,68]. Oncofetal reprogramming has been described within the TME for TAMs and ECs, yet it remains to be investigated whether cancer-associated fibroblasts (CAFs), T and B cells, NK cells and DCs also undergo oncofetal reprogramming. ICIs and anti-angiogenic molecules target receptors, ligands, cytokines, and chemokines that are crucial in the oncofetal reprogramming of FOLR2^+^ TAMs and PLVAP^+^ ECs and promotion of immunosuppression within the TME of HCC. Thus, it is possible that the oncofetal ecosystem plays a role in immunotherapy response both directly and indirectly through immunosuppression. It is important to note that the heterogeneity of the TME means that not all cells will display oncofetal reprogramming or the described receptors/ligands.

There is a current need for precision medicine with respect to immunotherapy; in HCC only a proportion of patients respond to immunotherapy (∼20–30%), treatment is expensive, and comes with considerable side effects in a significant proportion of patients [40]. Hence, elucidating the role of the oncofetal ecosystem in therapy response will pave the way for precision medicine, determining which treatment to give to which patient based on the composition of their tumor.

## Perspectives

- Epithelial cells are known to exhibit embryonic-like reprogramming in cancer. The concept of an oncofetal ecosystem in HCC was the first time fetal-like reprogramming of cells within the tumor microenvironment was observed.
- Oncofetal reprogramming orchestrates an immunosuppressive ecosystem in tumors. Therefore, the presence of oncofetal cells could predict immunotherapy response as well as provides a target for anti-cancer therapy.
- Determining an oncofetal score, a quantification of oncofetal cells within the TME, could predict tumor progression and/or therapy response in the clinic, thereby paving the way for precision medicine.

## Abbreviations

ALB: albumin
CAF: cancer-associated fibroblast
DC: dendritic cell
EC: endothelial cells
EMT: epithelial-to-mesenchymal transition
FOLR2: folate receptor beta
HBC: Hofbauer cells
HCC: hepatocellular carcinoma
ICI: immune checkpoint inhibitor
LSEC: liver sinusoidal endothelial cell
PLVAP: plasmalemma vesicle-associated protein
RECIST: response evaluation criteria in solid tumors
TAM: tumor-associated macrophages
TME: tumor microenvironment
TNF: tumor necrosis factor
Tregs: regulatory T cells
VEGF: vascular endothelial growth factor
